# MRI radiomic study on prediction of nonenlarged lymph node metastasis of rectal cancer: reduced field-of-view *versus* conventional DWI

**DOI:** 10.1186/s41747-025-00575-0

**Published:** 2025-03-22

**Authors:** Weinuo Qu, Jing Wang, Xuemei Hu, Yaqi Shen, Yang Peng, Daoyu Hu, Zhen Li

**Affiliations:** 1 Department of Radiology, Tongji Hospital, Tongji Medical College, Huazhong University of Science and Technology, Wuhan, P.R. China; 2 Department of Pediatrics, Tongji Hospital, Tongji Medical College, Huazhong University of Science and Technology, Wuhan, P.R. China

**Keywords:** Diffusion magnetic resonance imaging, Lymph nodes, Magnetic resonance imaging, Radiomics, Rectal neoplasms

## Abstract

**Background:**

Nonenlarged lymph node metastasis (NELNM) of rectal cancer is easily overlooked because these apparently normal lymph nodes are sometimes too small to measure directly using imaging techniques. Radiomic-based multiparametric imaging sequences could predict NELNM based on the primary lesion of rectal cancer. We aimed to study the performance of magnetic resonance imaging (MRI) radiomics derived from reduced field-of-view diffusion-weighted imaging (rDWI) and conventional DWI (cDWI) for the prediction of NELNM.

**Methods:**

A total of 86 rectal cancer patients (60 and 26 patients in training and test cohorts, respectively), underwent multiparametric MRI. Radiomic features were extracted from the whole primary lesion of rectal cancer segmented on T2-weighted imaging (T2WI), rDWI, and cDWI, both with *b*-value of 800 s/mm^2^ and apparent diffusion coefficient (ADC) maps from both DWI sequences (rADC and cADC). The radiomic models based on the above imaging methods were built for the assessment of NELNM status. Their diagnostic performances were evaluated in comparison with subjective evaluation by radiologists.

**Results:**

rADC demonstrated a significant advantage over subjective assessment in predicting NELNM in both training and test cohorts (*p* ≤ 0.002). In the test cohort, rADC exhibited a significantly higher area under the receiver operating characteristics curve than cADC, cDWIb800, and T2WI (*p* ≤ 0.020) in assessing NELNM for region-of-interest (ROI) delineation while excelling over rDWIb800 for prediction of NELNM (*p* = 0.0498).

**Conclusion:**

Radiomic features based on rADC outperformed those derived from T2WI and fDWI in predicting the NELNM status of rectal cancer, rADC was more advantageous than rDWIb800 in assessing NELNM.

**Relevance statement:**

Advanced rDWI excelled over cDWI in radiomic assessment of NELNM of rectal cancer, with the best performance observed for rADC, in contrast to rDWIb800, cADC, cDWIb800, and T2WI.

**Key Points:**

- rDWI, cDWI, and T2WI radiomics could help assess NELNM of rectal cancer.
- Radiomic features based on rADC outperformed those based on rDWIb800, cADC, cDWIb800, and T2WI in predicting NELNM.
- For rDWI radiomics, the ADC map was more accurate and reliable than DWI to assess NELNM for region of interest delineation.

**Graphical Abstract:**

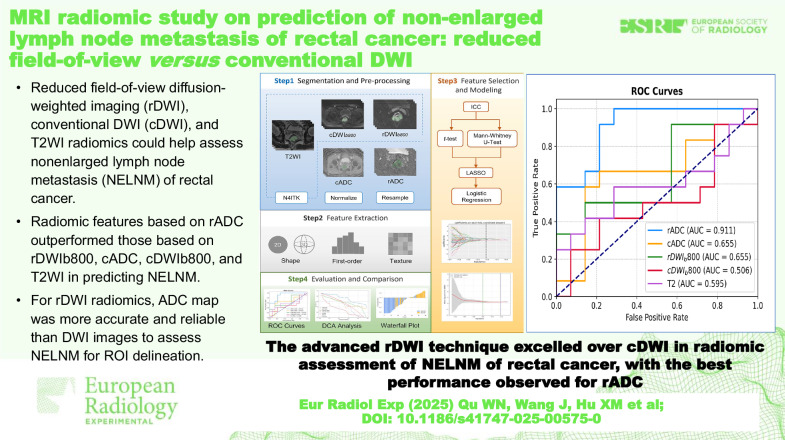

## Introduction

Lymph node (LN) metastasis is an important prognostic factor of rectal cancer [1]. There are two types of pelvic LNs closely associated with treatment planning and survival rate of rectal cancer, namely mesenteric LNs and lateral LNs [2]. Total mesorectal surgical excision is the standardized treatment for rectal cancer, including resection of the involved segment of the rectum, surrounding mesorectal structures, and mesenteric LNs [3]. Dissection of lateral LNs suspected to be metastatic should be performed in rectal cancer patients to avoid recurrence. However, accurately assessing LN metastasis preoperatively remains challenging, and extensive removal of pelvic LNs might result in unexpected complications like lymphatic and vascular damage, dysuria, or sexual dysfunction [4].

Magnetic resonance imaging (MRI) is routinely performed to reflect imaging features of rectal cancer with potential LN metastasis. In previous studies [5, 6], diagnosis of LN metastasis was based on size, shape, and intranodal characteristics. A short LN diameter of over 9 mm was often considered a sign of LN metastasis [6]. However, in the case of nonenlarged LNs, the conventional criteria for size and signal abnormalities may not be applicable, as they may appear similar to benign LNs for these characteristics [7]. Moreover, Langman et al [8] and Kasai et al [9] reported that the diameter for some of the LN metastasis of rectal cancer was not over 3 mm. Hence, it is clinically important to preoperatively assess nonenlarged LNs of rectal cancer with potential metastasis.

Diffusion-weighted imaging (DWI) MRI is mostly based on single-shot echo-planar imaging, which is often utilized for high signal-to-noise ratio and low motion artifacts sensitivity [10]. Nevertheless, the spatial resolution of conventional DWI (cDWI) is inherently limited, leading to suboptimal image quality that significantly hinders the detection of rectal lesions and the precise delineation of lesion margins. Moreover, this technique is vulnerable to distortions and blurring caused by field inhomogeneity [11].

To overcome these limitations, reduced field-of-view DWI (rDWI) has been introduced to improve image resolution and reduce distortions and artifacts [10, 12]. This technique incorporates a two-dimensional radiofrequency pulse into cDWI, effectively reducing the field of view and thereby mitigating susceptibility artifacts and geometric distortions [13]. Additionally, the application of an in-plane registration algorithm for motion compensation further improves image sharpness and the conspicuity of apparent diffusion coefficient (ADC) maps [11].

Radiomics is an emerging technique that enhances the utility of imaging data by extracting and analyzing radiologic information that cannot be detected by the naked eye [14]. Previous studies [15, 16] showed the potential of radiomics on T2-weighted imaging (T2WI) and cDWI for assessing rectal cancer. DWI-based radiomic analyses in relevant studies can be conducted using either high b-value DWI images or ADC maps [17, 18]. Yet, no studies indicated the differences between these two methods.

Therefore, in view of the above advantages of the rDWI technique, we assumed that in patients with rectal cancer, the diagnostic performance of radiomics of pelvic nonenlarged LNs could be better if based on rDWI than on cDWI, considering both the DWI images and ADC maps. In particular, we used rDWI for the prediction of nonenlarged lymph node metastasis (NELNM) compared with cDWI and high-resolution T2WI.

## Methods

### Study population

**Fig. 1:**
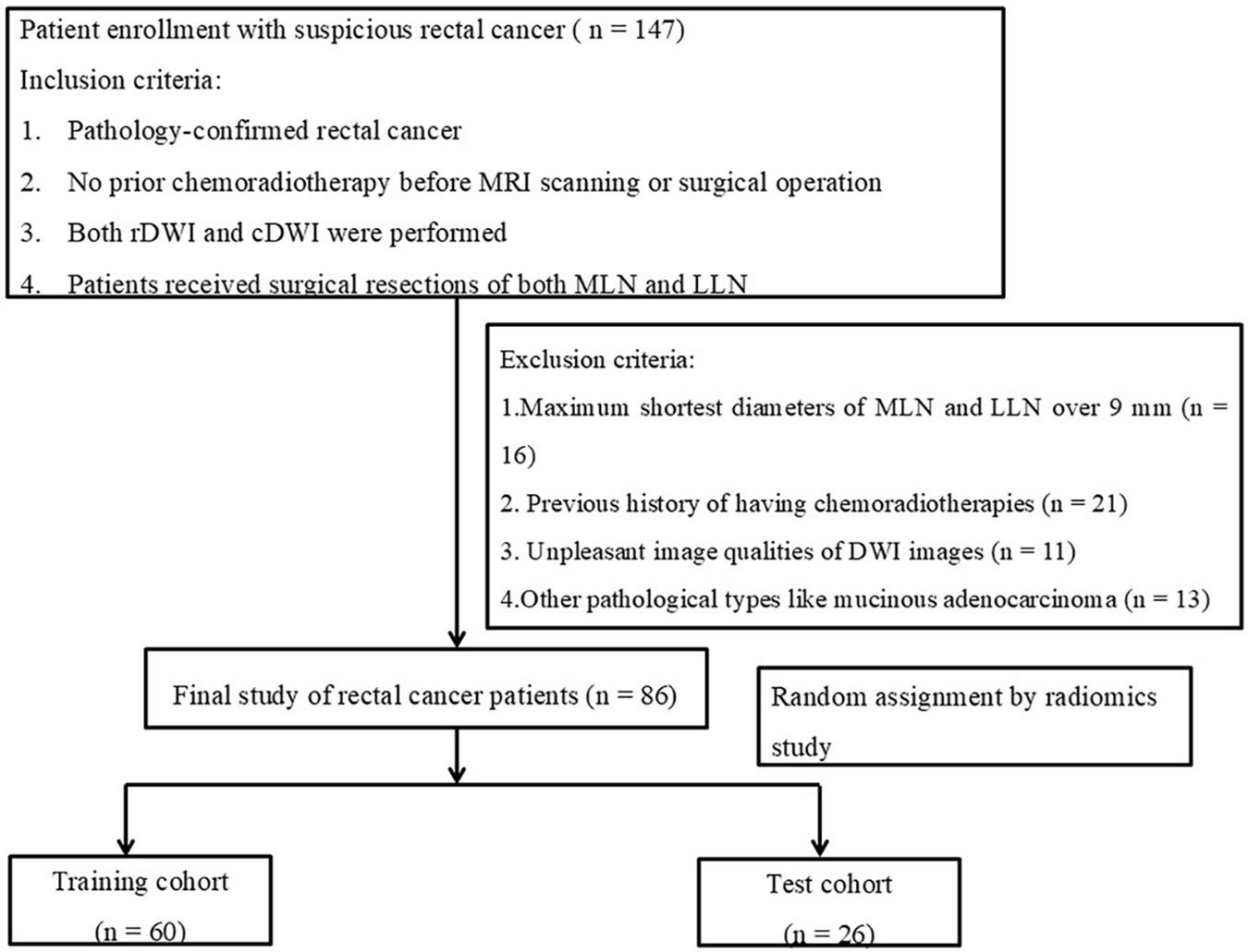
Flowchart of enrolled rectal cancer patients. MRI, Magnetic resonance imaging; rDWI, Reduced field-of-view diffusion-weighted imaging; cDWI, Conventional diffusion-weighted imaging; MLN, Mesenteric lymph node; LLN, Lateral lymph node; DWI, Diffusion-weighted imaging

This retrospective study was approved by the institutional review board with a waiver of informed consent. Patients with rectal lesions meeting the study criteria were identified from our hospital database, along with their corresponding clinical and imaging data. Between March 2016 and July 2021, a total of 147 patients were initially recruited. The inclusion criteria were as follows: (1) histopathological confirmation of rectal cancer via surgical resection or local biopsy; (2) no prior chemoradiotherapy before MR imaging or surgery; (3) availability of both rDWI and cDWI; and (4) surgical resection of both mesenteric LNs and lateral LNs. The exclusion criteria included following aspects: (1) high-resolution T2-weighted imaging (T2WI) showing mesenteric LNs or lateral LNs with a maximum short-axis diameter exceeding 9 mm (*n* = 16); (2) prior chemoradiotherapy (*n* = 21); (3) unpleasant DWI image quality due to severe motion artifacts or intestinal peristalsis (*n* = 11); and (4) pathological diagnosis of mucinous adenocarcinoma rather than rectal adenocarcinoma (*n* = 13). Finally, 86 patients were enrolled in this study for subsequent radiomic assessment (Fig. 1).

### MRI acquisition

Rectal cancer patients were scanned using a 3-T scanner (Discovery MR750, GE Healthcare, Waukesha, WI, USA) in supine position with a 32-channel phased array coil. To minimize motion artifacts, antiperistaltic agents were administered before scanning. The scanning protocol included T1-weighted, high-resolution not-fat-saturated T2WI, rDWI, and cDWI sequences with *b* values of 0 s/mm^2^ and 800 s/mm^2^ (Table 1).

**Table 1:** Sequence parameters

Parameters	T1WI	High-resolution T2WI	rDWI	cDWI
Sequence	FSE	FSE	EPI	EPI
Scanning plane	Oblique axial	Oblique axial	Oblique axial	Oblique axial
Repetition time (ms)	500	5,629	4,000	4,000
Echo time (ms)	75	85	75	75
Slice thickness (mm)	3	3	3	3
Interslice gap (mm)	1	0	0	0
Field of view (mm^2^)	380 × 380	200 × 200	200 × 100	400 × 400
Matrix (mm^2^)	320 × 224	448 × 314	128 × 64	160 × 128
*b*-value (s/mm^2^)	−	−	0, 800	0, 800
Bandwidth (kHz)	62.50	31.3	250	250
Number of excitations	2	4	12	12

*cDWI* Conventional DWI, *DWI* Diffusion-weighted imaging, *EPI* Echo planar imaging, *FSE* Fast spin-echo, *rDWI* Reduced field-of-view DWI

### Patient clinical characteristics

Clinical and demographic data, including age, gender, tumor size, tumor length, tumor location, maximum short-axis diameter of LNs, T stage, and tumor grade, were collected for all rectal cancer patients (Table 2).

**Table 2:** Patient characteristics of training and test cohorts

	Training cohort			Test cohort		
Characteristics	NELNM positive	NELNM negative	*p*-value	NELNM positive	NELNM negative	*p*-value
Age (years, mean ± SD)	51.70 ± 10.48	58.15 ± 11.30	**0.027**^a^	52.75 ± 11.36	60.57 ± 12.16	0.105^a^
Gender (%)						
Male	21 (77.8)	23 (69.7)	0.481^b^	10 (83.3)	5 (35.7)	**0.014**^**b**^
Female	6 (22.2)	10 (30.3)		2 (16.7)	9 (64.3)	
Operation (%)						
Miles	5 (18.5)	12 (36.4)	0.127^b^	2 (16.7)	3 (21.4)	0.759^b^
Non-miles	22 (81.5)	21 (63.6)		10 (83.3)	11 (78.6)	
Tumor location (%)						
Upper	8 (29.6)	7 (21.2)	0.108^b^	1 (8.3)	0	0.180^b^
Middle	13 (48.2)	10 (30.3)		8 (66.7)	6 (42.9)	
Lower	6 (22.2)	16 (48.5)		3 (25.0)	8 (57.1)	
Tumor diameter (mm, mean ± SD)	31.26 ± 6.37	30.35 ± 9.76	0.679^a^	42.62 ± 12.06	35.72 ± 9.61	0.118^a^
Tumor length (mm, mean ± SD)	49.04 ± 16.56	41.40 ± 16.83	0.083^a^	40.49 ± 10.53	36.64 ± 11.26	0.380^a^
MSDLN (mm, mean ± SD)	5.61 ± 1.48	4.33 ± 1.32	**0.000**^a^	4.51 ± 0.94	3.94 ± 0.82	0.109^a^
pT stage (%)						
T1–2	2 (7.4)	14 (42.4)	**0.002**^**b**^	0	8 (57.1)	**0.002**^**b**^
T3–4	25 (92.6)	19 (57.6)		12 (100.0)	6 (42.9)	
Tumor grade (%)						
High-intermediate	12 (44.4)	30 (90.9)	**0.000**^**b**^	6 (50.0)	14 (100.0)	**0.004**^**b**^
Low	15 (55.6)	3 (9.1)		6 (50.0)	0	

*MSDLN* Maximum shortest diameter of lymph node, *NELNM* Non-enlarged lymph node metastasis, *MSDLN* Maximum shortest diameter of lymph node, *SD* Standard deviation

Bold-type *p*-values indicate statistical significance (*p* < 0.050)

^a^ *p*-values indicate independent *t*-test or Mann–Whitney *U*-test according to the normal or non-normal data distribution

^b^ *p*-values indicate χ^2^ test

### Assessment of NELNM and tumor segmentation

Two radiologists (W.Q. and Y.P.) with 6 years and 10 years of experience in abdominal imaging, blinded to clinical history and pathological results, independently assessed the presence of NELNM. The diagnostic criteria for NELNM included: (1) LN short-axis diameter < 9 mm; (2) irregular morphology (*e.g.*, lobulated, spiculated, or indistinct borders); and (3) internal heterogeneous signal intensity.

The radiologists evaluated all rDWI and cDWI images, using T2WI as a reference, to identify visible nonenlarged LNs with potential metastasis. Discrepancies between the two radiologists were resolved through consensus discussion prior to the disclosure of pathological results.

The same two radiologists independently measured the maximum short-axis diameter of LNs and the maximum diameter of rectal cancer on axial high-resolution T2WI. All visible mesenteric and lateral LNs were measured twice, and patients with LNs exceeding 9 mm in short-axis diameter were excluded from further radiomic analysis. The radiologists, blinded to pathological and clinical information, independently delineated regions of interest (ROIs) on the primary rectal lesions. ROI delineations were performed on the following images: high-resolution non-fat-saturated T2WI, rDWI, and cDWI with a *b*-value of 800 s/mm²; ADC maps were obtained from rDWI and cDWI sequences. ROIs were manually drawn slice-by-slice along the tumor boundaries on the five image types (T2WI, cDWIb800, cADC, rDWIb800, and rADC), using axial non-fat-saturated T2WI as a reference. Care was taken to exclude areas with cystic changes, hemorrhage, or necrosis. All ROI delineations were performed using 3D-Slicer software (version 5.0.2, www.slicer.org).

### Image preprocessing

To improve the accuracy of subsequent quantitative analysis, N4ITK bias field correction, an ITK-implemented algorithm that addresses MRI intensity inhomogeneity via iterative B-spline approximation, was applied to T2WI, rDWI, and cDWI images using Python’s simple ITK package. All images were resampled to a uniform voxel size of 3 × 3 × 3 mm^3^ and discretized using a bin width of 25.

### Radiomic feature extraction

**Fig. 2:**
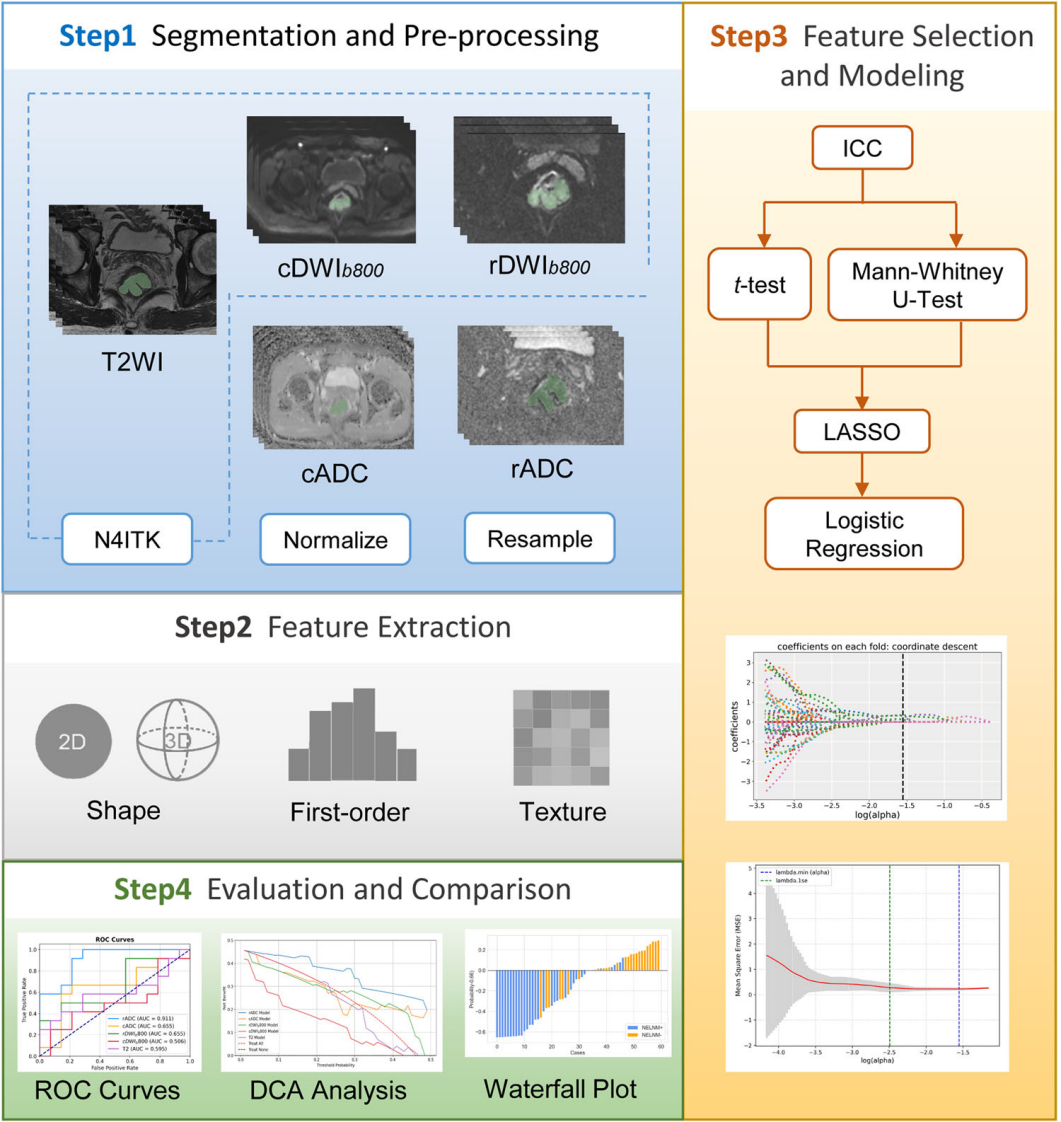
Study workflow. *cADC* and *rADC* indicated radiomic ROIs were delineated on ADC maps of corresponding DWI imaging, while *cDWI*_*b800*_ and *rDWI*_*b800*_ indicated radiomic ROIs were delineated on DWI images with *b* = 800 s/mm^2^. T2WI, T2 weighted imaging; ICC, Intraclass correlation coefficient; LASSO, Least absolute shrinkage and selection operator regression; ROC, Receiver operating characteristic curve; DCA, Decision curve analysis

We used Pyradiomics (http://pyradiomics.readthedocs.io/en/latest/index.html, version 3.1.0), an open-source Python package, to extract radiomics features from whole-tumor ROIs from high-resolution T2WI, rDWI, and cDWI sequences. The extracted radiomic features included the first-order statistics features, shape-based features, and texture features (gray level dependence matric, GLDM; gray level cooccurrence matrix, GLCM; gray level run length matrix, GLRLM; gray level size zone matrix, GLSZM; Neighboring Gray Tone Difference Matrix, NGTDM). Additionally, wavelet, Laplacian of Gaussian, and gradient filters were applied. For the Laplacian of Gaussian filter, sigma values were set to 3 and 5. A total of 1,130 radiomic features were extracted per lesion. The intraobserver and interobserver reproducibility of the radiomic features was assessed using the intraclass correlation coefficient. Features with values of intraclass correlation coefficient greater than 0.75 were selected for further analysis. Given the relatively small sample size, Intraclass correlation coefficient analysis was performed on the entire dataset to ensure feature robustness and consistency, thereby improving the generalizability of the findings. The study workflow is illustrated in Fig. 2.

### Radiomic feature selection and model construction

Patients were randomly divided into a training cohort (*n* = 60) and a test cohort (*n* = 26) at a ratio of 7:3. Feature selection was performed on the training cohort using Python (version 3.8, www.python.org). Radiomic features were initially screened using the student *t*-test or Mann–Whitney *U*-test, depending on the normality of data distribution as determined by the Kolmogorov–Smirnov test. The least absolute shrinkage and selection operator regression were then applied to reduce feature dimensionality. Logistic regression was used to construct radiomic models based on the selected features. Detailed information on the retained radiomic features for each modality is provided in Supplementary Table S1.

### Model evaluation and comparison

Receiver operating characteristic curves were generated to evaluate the diagnostic performance of the radiomic models in both the training and test cohorts. The area under the receiver operating characteristics curve (AUC), accuracy, sensitivity, specificity, positive predictive value, and negative predictive value are calculated to assess model performance. AUCs were compared for models derived from T2WI, rDWIb800, rADC maps, cDWIb800, and cADC maps. In addition, the AUC of subjective assessment for NELNM was compared with the radiomic models.

### Decision curve analysis (DCA)

DCA was conducted in the test cohort to evaluate the clinical utility of the radiomic models for predicting NELNM status in rectal cancer. The DCA was designed based on established methodologies [19, 20]. Given that LNs ≤ 5 mm account for approximately 53% of metastatic LNs in colorectal cancer, the risk threshold range for DCA was set at 0–50% [21].

### Pathological evaluation

All patients underwent total mesorectal excision, including removal of mesenteric and lateral LNs suspected of metastasis. Postoperatively, nonenlarged LNs were classified as NELNM positive (for metastasis) or NELNM negative (for metastasis) based on pathological reports, following the American Joint Committee on Cancer 8th edition TNM staging system for rectal cancer [22]. T stage and tumor grade were also recorded from pathological results.

### Statistical analysis

The Kolmogorov–Smirnov test was used to assess data normality. Quantitative clinical data were analyzed using the student *t*-test or Mann–Whitney *U*-test, while qualitative data were evaluated using the χ^2^ or Fisher exact test. The DeLong test was applied to compare AUCs among T2WI, DWI, and subjective assessment for NELNM. Statistical analyses were performed using SPSS (version 27.0, Armonk, NY, USA), MedCalc (version 12.7.0.0, Mariakerke, Belgium), and Python (version 3.8). A *p*-value lower than 0.05 was considered significant.

## Results

### Clinical characteristics of patients

The clinical characteristics of patients, stratified into NELNM positive and NELNM negative groups, are summarized in Table 2. Of the initial 147 patients, 86 met the inclusion criteria, while 61 were excluded based on the predefined exclusion criteria. A total of 784 nonenlarged LNs were harvested from these patients, with a size of 4.69 ± 1.40 mm (mean ± standard deviation), ranging from 2.32 mm to 8.38 mm. Among the 86 patients, 39 were classified as NELNM positive and 47 as NELNM negative. No significant differences were observed between the training and test cohorts in terms of surgical procedure, tumor location, tumor diameter, or tumor length (*p* = 0.108–0.759). However, the age and maximum short-axis diameter of LNs were significantly different in the training cohort for NELNM differentiation (*p* = 0.027 and *p* < 0.001, respectively), but not in the test cohort (*p* = 0.105 and *p* = 0.109, respectively). Both T stage and tumor grade showed significant differences in predicting NELNM in both cohorts (*p* = 0.000–0.004).

### Diagnostic performance of radiomic features from T2WI and DWI

**Fig. 3:**
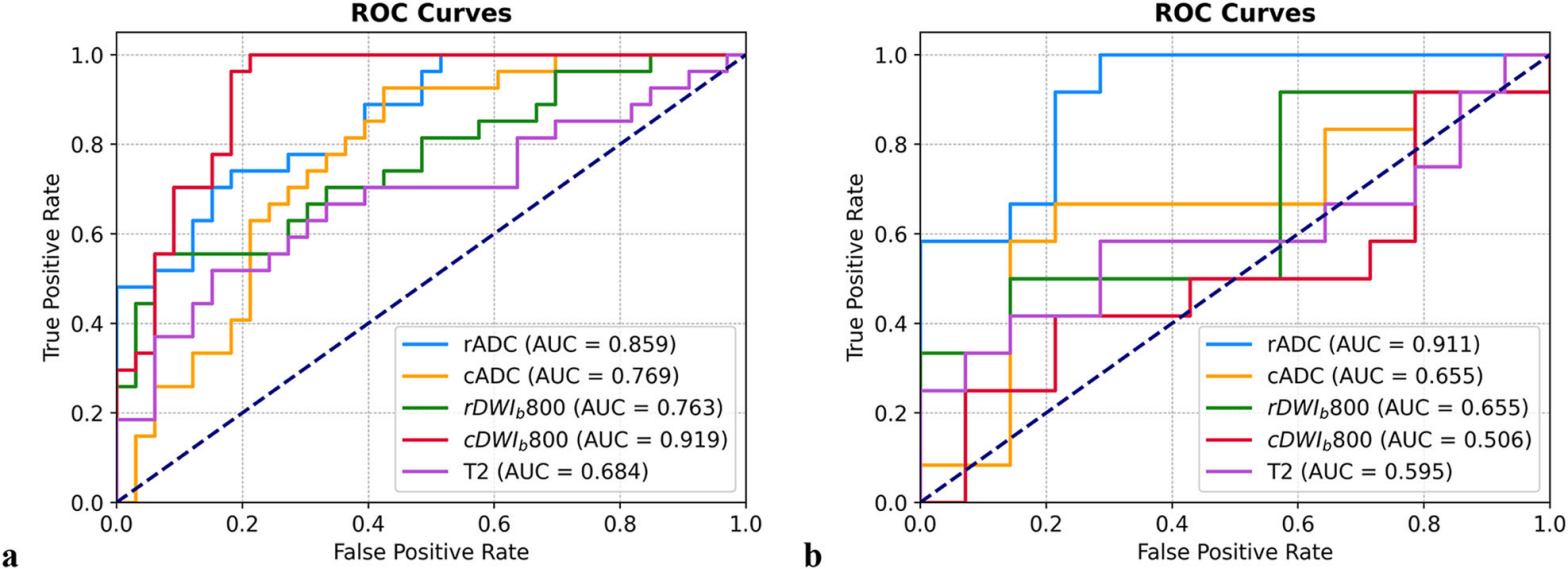
Diagnostic performances of radiomic models based on T2WI, rADC, cADC, rDWI_b800_, and cDWI_b800_ in the training cohort (**a**) and test cohort (**b**). *cADC* and *rADC* indicated radiomic ROIs were delineated on ADC maps of corresponding DWI imaging, while *cDWI*_*b80*0_ and *rDWI*_*b800*_ indicated radiomic ROIs were delineated on DWI images with *b* = 800 s/mm^2^. ROC, Receiver operating characteristic curve; AUC, Area under the curve

**Fig. 4:**
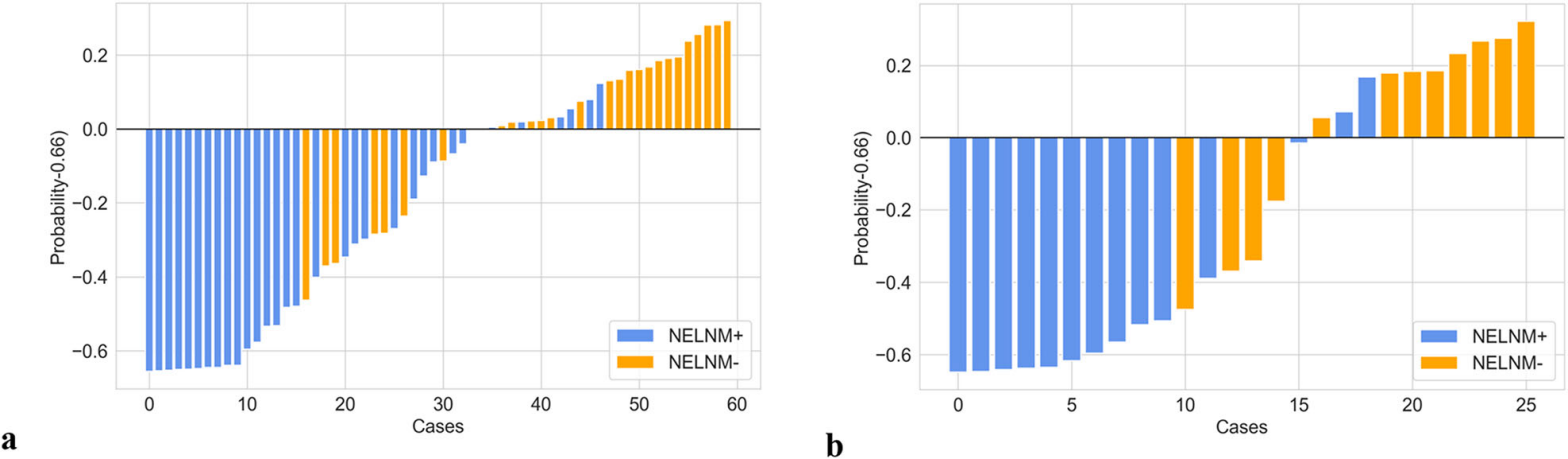
The waterfall images for displaying the probabilities calculated from the rADC model of the positive status of NELNM of rectal cancer in training cohort (**a**) and test cohort (**b**). NELNM, Nonenlarged lymph node metastasis

Receiver operating characteristic curves and AUC values were calculated to differentiate between NELNM positive and NELNM negative cases (Fig. 3). Sensitivity, specificity, positive predictive value, and negative predictive value were derived for T2WI, rDWIb800, rADC, cDWIb800, and cADC models (Table 3). Significant differences in performance were observed between T2WI and rADC in both the training and test cohorts (*z* = 1.993, *p* = 0.046 and *z* = 2.330, *p* = 0.020, respectively), while cDWIb800 showed a significant difference only in the training cohort (*z* = 2.850, *p* = 0.004). Among rDWI and cDWI models, rADC demonstrated superior performance compared to cDWIb800, cADC, and rDWIb800 in the test cohort (*z* = 1.965, *p* = 0.0495; *z* = 2.948, *p* = 0.003; and *z* = 1.961, *p* = 0.0498, respectively). Additionally, cDWIb800 outperformed cADC and rDWIb800 in the training cohort (*z* = 2.135, *p* = 0.033 and *z* = 2.106, *p* = 0.035, respectively). No other significant differences were observed among the remaining DWI models (*p* = 0.220–1.000) (Table 4 and Fig. 4).

**Table 3:** Diagnostic performance of radiomic features from T2WI and DWI of rectal cancer

	AUC (95% CI)		Sensitivity		Specificity		PPV		NPV	
Sequences	Train cohort	Test cohort	Train cohort	Test cohort	Train cohort	Test cohort	Train cohort	Test cohort	Train cohort	Test cohort
T2WI	0.684 (0.520, 0.825)	0.595 (0.338, 0.812)	0.667 (0.481, 0.842)	0.583 (0.286, 0.833)	0.636 (0.462, 0.789)	0.429 (0.167, 0.688)	0.600 (0.407, 0.759)	0.467 (0.214, 0.714)	0.700 (0.533, 0.867)	0.545 (0.250, 0.833)
cADC	0.769 (0.638, 0.882)	0.655 (0.420, 0.881)	0.630 (0.429, 0.812)	0.583 (0.300, 0.875)	0.788 (0.643, 0.909)	0.857 (0.636, 1.000)	0.708 (0.500, 0.875)	0.778 (0.500, 1.000)	0.722 (0.571, 0.867)	0.706 (0.467, 0.929)
cDWI_b800_	0.919 (0.834, 0.979)	0.506 (0.261, 0.758)	0.852 (0.708, 0.969)	0.500 (0.222, 0.800)	0.818 (0.675, 0.941)	0.500 (0.214, 0.778)	0.793 (0.625, 0.931)	0.462 (0.200, 0.750)	0.871 (0.759, 0.973)	0.538 (0.250, 0.833)
rADC	0.859 (0.754, 0.939)	0.911 (0.781, 1.000)	0.630 (0.429, 0.812)	0.667 (0.400, 0.917)	0.788 (0.643, 0.909)	0.786 (0.556, 1.000)	0.708 (0.500, 0.875)	0.727 (0.444, 1.000)	0.722 (0.571, 0.867)	0.733 (0.500, 0.938)
rDWI_b800_	0.763 (0.632, 0.873)	0.655 (0.400, 0.875)	0.704 (0.500, 0.857)	0.583 (0.300, 0.846)	0.636 (0.467, 0.788)	0.429 (0.167, 0.727)	0.613 (0.429, 0.778)	0.467 (0.222, 0.733)	0.724 (0.560, 0.879)	0.545 (0.222, 0.833)

cADC and rADC indicated radiomic ROIs were delineated on ADC maps of corresponding DWI imaging, while cDWI_b800_ and rDWI_b800_ indicated radiomic ROIs were delineated on DWI images with *b* = 800 s/mm^2^

*p*-value lowers than 0.050 indicate statistical significance

*ADC* Apparent diffusion coefficient, *AUC* Area under the receiver operating characteristics curve, *cDWI* Conventional DWI, *CI* Confidence interval, *DWI* Diffusion-weighted imaging, *NPV* Negative predictive value, *PPV* Positive predictive value, *rDWI* Reduced field-of-view DWI

**Table 4:** Comparison of performances of radiomic features from T2WI and DWI of rectal cancer

AUC comparison	Training cohort		Test cohort	
	z-value	*p*-value	z-value	*p*-value
T2WI *versus* cADC	0.886	0.376	0.345	0.730
T2WI *versus* cDWI_b800_	2.850	**0.004**	-0.509	0.611
T2WI *versus* rADC	1.993	**0.046**	2.330	**0.020**
T2WI *versus* rDWI_b800_	0.817	0.414	0.347	0.729
rADC *versus* cADC	1.177	0.239	1.965	**0.0495**
rDWI_b800_ *versus* cDWI_b800_	-2.135	**0.033**	0.858	0.391
cADC *versus* cDWI_b800_	-2.106	**0.035**	0.855	0.393
rADC *versus* rDWI_b800_	1.226	0.220	1.961	**0.0498**
rADC *versus* cDWI_b800_	-1.030	0.303	2.948	**0.003**
cADC *versus* rDWI_b800_	0.0684	0.946	0.000	1.000

Comparisons of diagnostic performances between different radiomic features from T2WI and DWI using ADC map-based and DWI image-based methods were performed by the Delong test. Bold type *p*-values indicate statistical significance. cADC and rADC indicated radiomic ROIs were delineated on ADC maps of corresponding DWI imaging, while cDWI_b800_ and rDWI_b800_ indicated radiomic ROIs were delineated on DWI images with *b* = 800 s/mm^2^

*ADC* Apparent diffusion coefficient, *AUC* Area under the receiver operating characteristics curve, *cDWI* Conventional DWI, *DWI* Diffusion-weighted imaging, *rDWI* Reduced field-of-view DWI

### Comparison of imaging-based radiomic models with subjective assessment of NELNM

The AUC values for subjective assessment of NELNM based on rDWI and cDWI ranged from 0.405 to 0.503, with the highest value (0.503) observed for rDWI in the training cohort. Significant differences were noted between all imaging-based radiomic models and subjective assessment in the training cohort (*z* = 2.634–4.867, *p* = 0.000–0.008). In the test cohort, only the rADC model showed a significant difference compared to subjective assessment (*z* = 3.041, *p* = 0.002) (Table 5).

**Table 5:** Subjective assessment of NELNM of rectal cancer based on cDWI and rDWI in comparison with radiomic performances

Subjective assessment	cDWI		rDWI	
	Training cohort	Test cohort	Training cohort	Test cohort
Accuracy	29/60	12/26	31/60	13/26
SAUC (95% CI)	0.488 (0.340, 0.636)	0.405 (0.184, 0.626)	0.503 (0.355, 0.651)	0.482 (0.256, 0.709)

AUC Comparison	Training cohort		Test cohort	
	*z*-value	*p*-value	*z*-value	*p*-value
cADC *versus* SAUC	2.658	**0.008**	0.362	0.717
cDWI_b800_ *versus* SAUC	4.867	**0.000**	-0.528	0.597
rADC *versus* SAUC	4.031	**0.000**	3.041	**0.002**
rDWI_b800_ *versus* SAUC	2.634	**0.008**	0.821	0.412

Accuracy indicated the correct number of diagnoses made by two radiologists in consensus before knowing the final results, divided by the total number of cases for diagnosis of NELNM

cADC and rADC indicated that radiomic ROIs were delineated on ADC maps of corresponding DWI images, while cDWI_b800_ and rDWI_b800_ indicate that radiomic ROIs were delineated on DWI images with *b* = 800 s/mm^2^

Bold type *p*-values indicate statistical significance

*AUC* Area under the curve, *cDWI* Conventional diffusion-weighted imaging, *rDWI* Reduced field-of-view diffusion-weighted imaging, *SAUC* Area under the curve made by subjective assessment

### DCA

**Fig. 5:**
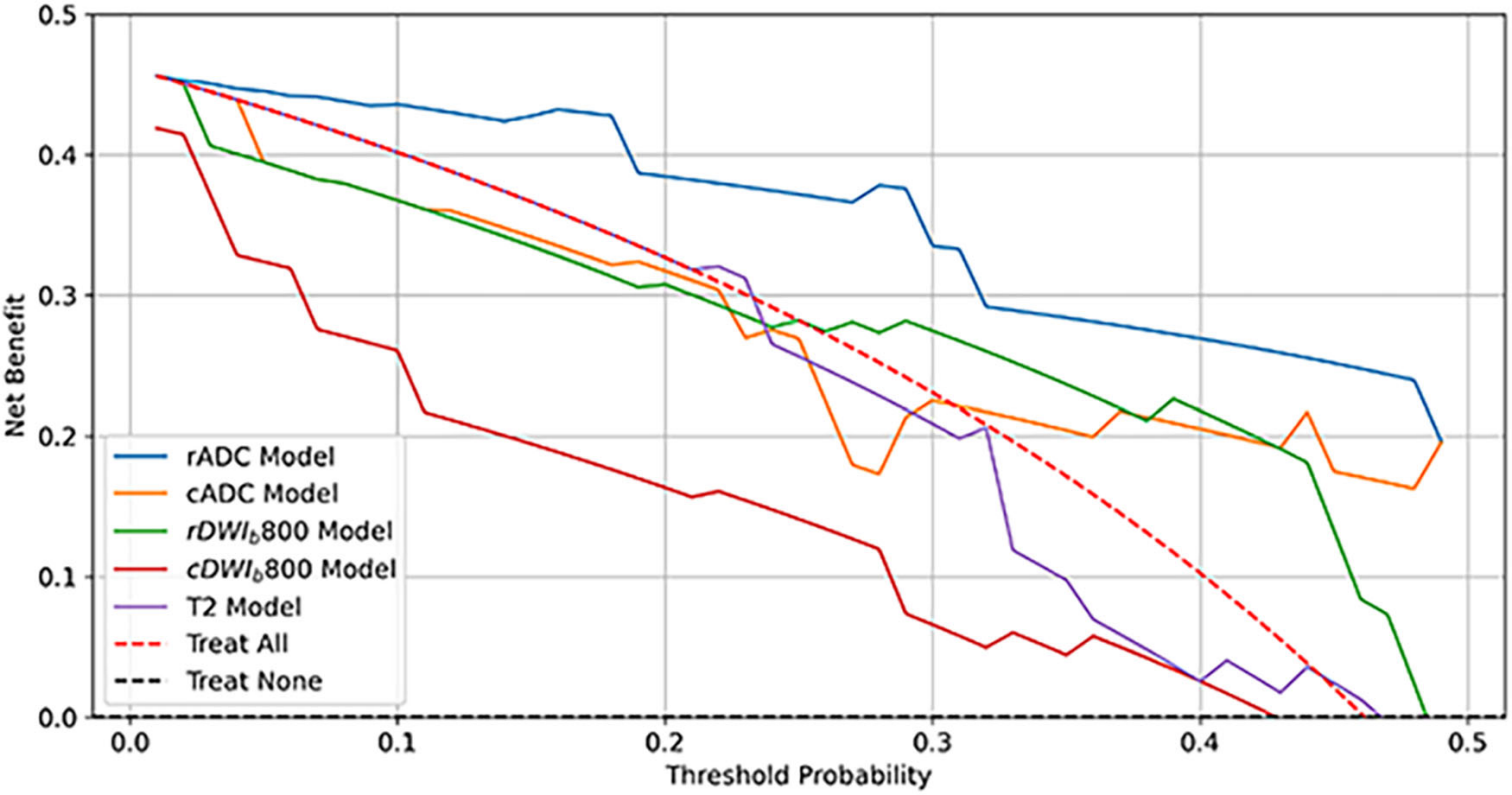
DCA curves of different radiomic models applied on T2WI, rADC, cADC, rDWI_b800_, and cDWI_b800_ in the test cohort. *cADC* and *rADC* indicated radiomic ROIs were delineated on ADC maps of corresponding DWI imaging, while *cDWI*_*b800*_ and *rDWI*_*b800*_ indicated radiomic ROIs were delineated on DWI images with *b* = 800 s/mm^2^. DCA, Decision curve analysis

DCA for radiomic models based on T2WI, rDWI, and cDWI is presented in Fig. 5. Across the threshold probability range, the rADC model demonstrated the highest net benefit, outperforming all other models. In contrast, cDWIb800 yielded a lower net benefit than treating all patients as NELNM positive. At a threshold probability of 18%, rADC achieved a net benefit of 0.43, suggesting its reliability for predicting NELNM in rectal cancer. The T2WI, rDWIb800, and cADC models showed limited or inconsistent net benefit within threshold ranges of 21–23%, 28–47%, and 33–50%, respectively.

## Discussion

The LN status is a critical factor in treatment planning and prognosis for rectal cancer [23]. In clinical practice, nonenlarged mesenteric and lateral LNs are often overlooked due to their resemblance to normal LNs and the limitations of conventional imaging techniques [24]. Moreover, some LNs are too small to be reliably detected or may lack sufficient imaging information. To address these challenges, we investigated the use of advanced DWI combined with radiomics to predict NELNM status based on primary rectal cancer lesions. Furthermore, we compared the performance of radiomic features extracted from DWI images and ADC maps to determine the optimal approach for radiomic analysis in rectal cancer. Our results demonstrated that the rADC model outperformed rDWIb800, cDWIb800, cADC, and T2WI in predicting NELNM status. Notably, rADC-based radiomics also showed superior performance compared to subjective assessments by radiologists. To our knowledge, this is the first study to evaluate NELNM in rectal cancer using DWI-based radiomics by comparison of different ROI delineation approaches for both DWI images and ADC maps.

Our findings indicated that radiomic models derived from rDWI outperformed those based on cDWI and T2WI in predicting NELNM. Although both rDWI and cDWI are DWI sequences, they differ in their underlying mechanisms. cDWI, typically using single-shot echo-planar imaging, is prone to image artifacts and limited spatial resolution [10, 25]. In contrast, rDWI incorporates a two-dimensional radiofrequency pulse, reducing the field-of-view along the phase-encoding direction and improving image quality without extending scan time [26]. This improvement allowed for more accurate ROI delineation and, consequently, more reliable radiomic feature extraction compared to cDWI.

Interestingly, rADC demonstrated superior performance over rDWIb800 in the radiomic analysis of NELNM. While ROIs are typically delineated on either ADC maps or high *b*-value DWI images, no prior studies have directly compared these approaches for NELNM evaluation in rectal cancer. High *b*-value DWI images provide high contrast by reflecting water molecule diffusion restriction in tumor tissues, with high signal areas indicating regions of restricted diffusion [27, 28]. However, DWI images are susceptible to T2 shine-through effects and artifacts, which can compromise the accurate visualization of rectal lesions [29]. In contrast, ADC maps eliminate the T2 shine-through effect and reduce artifacts, offering a more quantitative and reliable representation of diffusion [30]. This might explain the superior performance of rADC over rDWIb800. Conversely, cADC and cDWIb800 models performed poorly in predicting NELNM, likely due to the inherent limitations of cDWI compared to rDWI.

Our study indicated that rADC-based radiomics significantly outperformed the subjective assessment of NELNM. Nonenlarged LNs often resemble normal LNs in morphology, making it challenging to accurately assess their metastatic status based on size, shape, and internal homogeneity [7, 31, 32]. In contrast, DWI-based radiomics provides a more objective and comprehensive approach to predicting NELNM status [33]. Since the small size of LNs precluded direct ROI delineation, we focused on whole-tumor ROIs from primary rectal cancer lesions, demonstrating the potential of DWI radiomics for NELNM assessment.

Several limitations of our study must be taken into account. First, the sample size was relatively small, and future studies with larger cohorts are needed to validate our findings. Second, the retrospective design may introduce selection bias. Third, we excluded patients with enlarged LNs to focus on the performance of DWI radiomics in predicting NELNM. Future studies should include patients with both enlarged and nonenlarged LNs for a more comprehensive evaluation. Fourth, some pathologically confirmed metastatic LNs were too small to be reliably detected or distinguished from normal LNs on DWI and T2WI images. Consequently, a node-by-node correlation between imaging findings and pathological results was not feasible. Finally, all data were obtained at a single institution, and multicenter studies are necessary to enhance the generalizability of our results.

In conclusion, radiomics based on T2WI and DWI could help predict NELNM in rectal cancer. The rADC model outperformed rDWIb800, cADC, cDWIb800, and T2WI in assessing NELNM status. Our findings suggested that ADC maps were more reliable than DWI images for constructing rDWI-based radiomic models to predict NELNM status in rectal cancer.

## Supplementary information

**Additional file 1: Table S1.** Radiomic features remained after the selection.

## Data Availability

The data could be requested from the corresponding author upon reasonable request.

## Abbreviations

ADC: Apparent diffusion coefficient
AUC: Area under the receiver operating characteristics curve
cDWI: Conventional DWI
DCA: Decision curve analysis
DWI: Diffusion-weighted imaging
LN: Lymph node
MRI: Magnetic resonance imaging
NELNM: Nonenlarged lymph node metastasis
rDWI: Reduced field-of-view DWI
ROI: Region-of-interest
T2WI: T2-weighted imaging
