# Supplementary material for: MRI radiomic study on prediction of nonenlarged lymph node metastasis of rectal cancer: reduced field-of-view versus conventional DWI
Source: Eur Radiol Exp. 2025 Mar 22;9:34. doi: 10.1186/s41747-025-00575-0 (PMC11929653; doi:10.1186/s41747-025-00575-0)
Supplement: Supplementary file 1 — Additional file 1: Table S1. Radiomic features remained after the selection. [file 41747_2025_575_MOESM1_ESM.pdf]

MRI radiomic study on prediction of nonenlarged lymph node metastasis of rectal cancer: reduced field-of-view versus conventional DWI

ELECTRONIC SUPPLEMENTARY MATERIAL

Eur Radiol Exp (2025) Qu WN, Wang J, Hu XM, et al.

Table S1. Radiomic features remained after selection

Modality /Methods	Radiomic features
T2WI	original_shape_Maximum2DdiameterRow
	original_shape_Sphericity
	gradient_glrlm_RunLengthNonUniformity
	original_firstorder_InterquartileRange
	wavelet-LLH_firstorder_90Percentile
	wavelet-LLH_firstorder_InterquartileRange
cADC	wavelet-LLH_glcm_Imc2
	wavelet-HLL_firstorder_Maximum
	wavelet-LLL_firstorder_Skewness
	wavelet-LLL_glrlm_RunLengthNonUniformity
	original_shape_Sphericity
	original_glszm_ZonePercentage
	wavelet-LLH_firstorder_RootMeanSquared
	wavelet-LLH_glcm_Imc2
	wavelet-LLH_glszm_LargeAreaHighGrayLevelEmphasis
$cDWI_{b800}$	wavelet-LHL_glcm_ClusterProminence
	wavelet-LHL_glszm_ZoneEntropy
	wavelet-HLL_firstorder_90Percentile
	wavelet-HLL_firstorder_Entropy
	wavelet-HLL_glrlm_HighGrayLevelRunEmphasis
	wavelet-HLL_ngtdm_Contrast
	log-sigma-3-mm-3D_firstorder_Skewness
	original_shape_MinorAxisLength
	original_firstorder_InterquartileRange
rADC	original_gldm_SmallDependenceHighGrayLevelEmphasis
	log-sigma-3-mm-3D_glrlm_GrayLevelNonUniformityNormalized
	log-sigma-5-mm-3D_glrlm_GrayLevelNonUniformityNormalized
	wavelet-HLL_glrlm_ShortRunHighGrayLevelEmphasis
	wavelet-LLH_glszm_GrayLevelNonUniformity
$rDWI_{b800}$	wavelet-LHH_glszm_LargeAreaLowGrayLevelEmphasis
	gradient_glszm_ZonePercentage

cADC and rADC indicated radiomic ROIs were delineated on ADC maps of corresponding DWI imaging, while $cDWI_{b800}$ and $rDWI_{b800}$ indicated radiomic ROIs were delineated on DWI images with *b* = 800 s/ mm^2. Wavelet-filtered radiomic features were derived from the decomposition of images into different frequency sub-bands. The acronyms (e.g., HLL, LHL) denote the sequence of high-pass (H) and low-pass (L) filtering applied at each decomposition level. 'H' represents high-pass filtering (capturing high-frequency details), while 'L' represents low-pass filtering (capturing low-frequency smooth components). For example, 'HLL' indicates high-pass filtering in the first dimension and low-pass filtering in the second and third dimensions.

glcm gray level co-occurrence matrix, *gldm* gray level dependence matrix, *glrlm* gray level run length matrix, *glszm* gray level size zone matrix, *ngtdm* neighboring gray tone difference matrix.
